# Decreased small mammal and on-host tick abundance in association with invasive red imported fire ants (*Solenopsis invicta*)

**DOI:** 10.1098/rsbl.2016.0463

**Published:** 2016-09

**Authors:** Adrian A. Castellanos, Matthew C. I. Medeiros, Gabriel L. Hamer, Michael E. Morrow, Micky D. Eubanks, Pete D. Teel, Sarah A. Hamer, Jessica E. Light

**Affiliations:** 1 Department of Wildlife and Fisheries Sciences, Texas A&M University, College Station, TX, USA; 2 Department of Entomology, Texas A&M University, College Station, TX, USA; 3 Pacific Biosciences Research Center, University of Hawai'i at Mãnoa, Honolulu, HI, USA; 4 Attwater Prairie Chicken National Wildlife Refuge, Eagle Lake, TX, USA; 5 Department of Veterinary Integrative Biosciences, Texas A&M University, College Station, TX, USA

**Keywords:** ecology, species interactions, vector, invasive species, tick-borne pathogens

## Abstract

Invasive species may impact pathogen transmission by altering the distributions and interactions among native vertebrate reservoir hosts and arthropod vectors. Here, we examined the direct and indirect effects of the red imported fire ant (*Solenopsis invicta*) on the native tick, small mammal and pathogen community in southeast Texas. Using a replicated large-scale field manipulation study, we show that small mammals were more abundant on treatment plots where *S. invicta* populations were experimentally reduced. Our analysis of ticks on small mammal hosts demonstrated a threefold increase in the ticks caught per unit effort on treatment relative to control plots, and elevated tick loads (a 27-fold increase) on one common rodent species*.* We detected only one known human pathogen (*Rickettsia parkeri*), present in 1.4% of larvae and 6.7% of nymph on-host *Amblyomma maculatum* samples but with no significant difference between treatment and control plots. Given that host and vector population dynamics are key drivers of pathogen transmission, the reduced small mammal and tick abundance associated with *S. invicta* may alter pathogen transmission dynamics over broader spatial scales.

## 1. Introduction

Invasive species can directly or indirectly alter vector-borne disease systems by changing the abundance of, or interactions between, vectors and their hosts. Previous studies have most commonly implicated the invader in altering species relationships in ways that support vector-borne pathogen transmission and, therefore, increase disease risk. For example, a widespread, invasive shrub increases human risk of ehrlichiosis because it provides habitat for deer that host infected ticks [1], and densities of ticks and tick hosts were greatest in areas that had been invaded by the causative agent of sudden oak death [2]. By contrast, with few exceptions (e.g. [3]), invasive species have less frequently been implicated in the reduction of infectious disease transmission. However, invasive host species may dilute vector-borne disease risk consistent with the dilution effect hypothesis [4]. For example, infection of native mice with flea-transmitted *Bartonella* species was reduced with increasing densities of introduced voles [5].

Here, we investigate the potential impact of the invasive red imported fire ant (*Solenopsis invicta*) on tick, small mammal and pathogen communities in southeast Texas. Ticks and small mammals transmit and maintain numerous zoonotic pathogens that are significant public health concerns. *Solenopsis invicta* are known to predate small mammals [6], and their presence is associated with changes in mammal foraging activity [7] and habitat selection [8] possibly mediated by changes in food resources [9]. *Solenopsis invicta* are also associated with reductions in tick populations [10,11], although effects vary between tick species [10]. Using a large-scale manipulative experiment to reduce *S. invicta* populations across an area of historic invasion, we expected that *S. invicta* predation and avoidance behaviour by mammals and ticks would lead to decreased mammal, tick and pathogen abundance in plots where *S. invicta* were in high density relative to treatment plots where *S. invicta* were experimentally suppressed.

## 2. Material and methods

The manipulative experiment occurred at two field sites separated by over 160 km in southeast Texas: Attwater Prairie Chicken National Wildlife Refuge (APCNWR) and a private ranch in Goliad County (GRR). Each field site was partitioned into two treatment plots and two control plots. Treatment plots were chemically treated with Extinguish Plus™ (Central Life Sciences, Schaumburg, IL, USA) for *S. invicta* suppression as part of an existing management plan for Attwater's prairie-chicken (*Tympanuchus cupido attwateri*) [12]; control plots were not treated. Efficacy of the treatment was monitored by setting out fatty lures in treatment and control plots ([12]; see the electronic supplementary material).

Small mammals and their attached ticks were collected using seed-baited Sherman live traps (H.B. Sherman Traps, Tallahassee, FL, USA). Three line transects (approx. 20 m apart), each with 20 traps spaced 10 m apart, were spread across each of the four plots at both field sites, resulting in a total of 60 traps per plot and 240 traps per site. Small mammal trapping was conducted for two consecutive nights each month (APCNWR: trapping occurred from June 2013 until September 2014; GRR: October 2013 until July 2014, with the exception of January 2014). All captured mammals were marked with an ear tag, identified to species and inspected for ticks, which were removed, identified and stored in 70% ethanol. Off-host tick presence was assessed via drag sampling (see the electronic supplementary material). On-host ticks were tested for infection with microbes in the genera *Rickettsia* and *Borrelia* (see the electronic supplementary material).

We used general linear mixed models assuming a negative binomial error distribution to analyse counts of mammals and on-host ticks across treatment and control plots. We used a zero-inflated (ZI) model if it fit the data better (i.e. lower Akaike information criteria) than the same model that did not account for ZI. All models were implemented in program R (v. 3.2.2) in the package glmmADMB (v. 0.8.3.2). Site (two levels, APCNWR and GRR) and season (four levels, spring = March to May; summer = June to August; autumn = September to November; winter = December to February) were added to models as random intercepts (mammal abundance was spatio-temporally heterogeneous throughout the study; see the electronic supplementary material). Sampling effort (effective trap nights) per transect was included in the model using the offset function. Significance of all treatment coefficients was assessed through a log-likelihood ratio test of nested models assuming a *χ*^2^-distribution. Association between pathogen infection of ticks (larval pools, larval individuals, and nymphs analysed separately) and *S. invicta* treatment was tested with a Fisher's exact test.

## 3. Results

**Figure 1.**
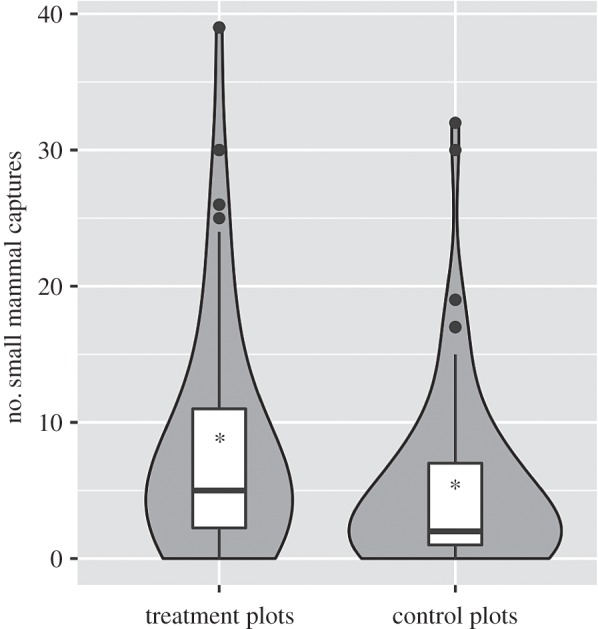
A violin/box plot hybrid demonstrating the number of small mammal captures on treatment and control plots. Asterisks denote means.

The majority (64.1%) of small mammals captured were from *S. invicta*-suppressed treatment plots (table 1; figure 1; electronic supplementary material, figure 1). Our model predicted a 1.8-fold increase in the total number of small mammals captured per unit effort on treatment relative to control plots (*p* < 0.001). The effect was consistent among the three most commonly sampled mammal species (*Sigmodon hispidus*, *Baiomys taylori* and *Reithrodontomys fulvescens*). Our model predicted a 2.0-fold increase in *S. hispidus* captured on treatment relative to control plots (*p* < 0.001). Effect sizes were slightly lower for *B. taylori* (1.4-fold increase on treatment plots, *p* = 0.01) and *R. fulvescens* (1.4-fold increase on treatment plots, *p* = 0.05).

**Table 1.** Mammal captures presented by species and plot type at both Attwater Prairie Chicken National Wildlife Refuge (APCNWR) and a private ranch in Goliad County, Texas (GRR). (Number of captures and percentage of total captures per site (in parentheses) are indicated for each species across site and plot type. Treatment plots are those that were treated with Extinguish Plus™ to suppress red imported fire ants.)

species	APCNWR treatment	APCNWR control	GRR treatment	GRR control	total
*Sigmodon hispidus* (hispid cotton rat)	354 (68.5%)	163 (31.5%)	11 (100%)	0 (0%)	528
*Baiomys taylori* (northern pygmy mouse)	127 (62.0%)	78 (38.0%)	107 (65.6%)	56 (34.4%)	368
*Reithrodontomys fulvescens* (fulvous harvest mouse)	69 (51.9%)	64 (48.1%)	63 (84.0%)	12 (16.0%)	208
*Chaetodipus hispidus* (hispid pocket mouse)	12 (42.9%)	16 (57.1%)	9 (56.3%)	7 (43.7%)	44
*Peromyscus leucopus* (white-footed mouse)	0 (0%)	27 (100%)	10 (90.9%)	1 (9.1%)	38
*Cryptotis parva* (least shrew)	0 (0%)	1 (100%)	6 (66.7%)	3 (33.3%)	10
*Perognathus merriami* (Merriam's pocket mouse)	0 (0%)	0 (0%)	0 (0%)	1 (100%)	1
*Oryzomys palustris* (marsh rice rat)	0 (0%)	1 (100%)	0 (0%)	0 (0%)	1
total	562	350	206	80	1198

**Figure 2.**
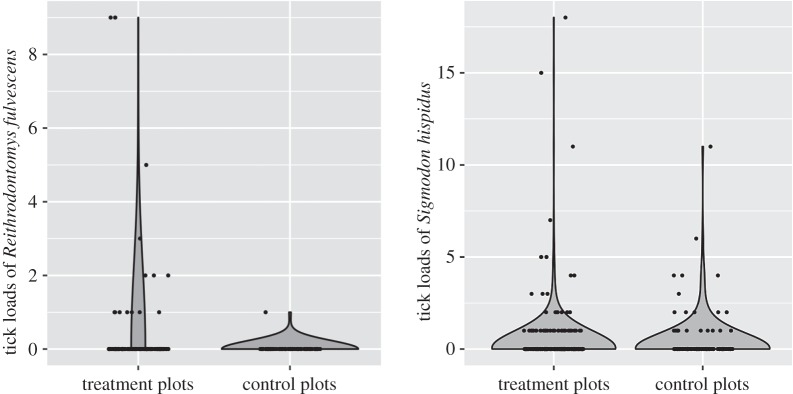
A violin plot demonstrating the probability density of tick loads on two species of small mammals. Dots represent actual observations, jittered horizontally to better demonstrate sample sizes.

Ninety-eight mammals (8.7% of captures) were parasitized by a total of 237 ticks, including 142 larvae and 95 nymphs (electronic supplementary material, tables S2 and S3). Nearly all ticks were *Ambylomma maculatum* (99.6%) with the exception of one nymphal *Ixodes scapularis* (0.4%). The rodent species most heavily parasitized by ticks were *S. hispidus* (15.6% of total captures), *Chaetodipus hispidus* (7.7%), *R. fulvescens* (7.7%) and *B. taylori* (1.4%). Our model predicted a threefold increase in the number of on-host ticks caught per unit effort on treatment relative to control plots (*p* = 0.01). When the number of rodents captured during a sampling night was included in the model with the offset function, the model still predicted an increase in the number of ticks on treatment plots, but this effect was no longer significant (*p* = 0.45). This suggests that the effect of a greater number of on-host ticks on treatment plots was primarily driven by an increased capture rate of small mammals along treatment transects. To directly investigate tick loads across treatment and control plots, we modelled the number of ticks per host individual in *S. hispidus* and *R. fulvescens*, two well-sampled (*N* = 482 and 195, respectively) and highly parasitized species in our data. Tick loads did not vary significantly across plots in *S. hispidus* (*p* = 0.90, figure 2), possibly due to demographic effects that resulted after an explosive increase in the population (see the electronic supplementary material). However, our model predicted a 27-fold increase in the tick loads on *R. fulvescens* on treatment relative to control plots (*p* = 0.003; figure 2). Drag sampling of 30 200 m^2^ of vegetation resulted in the collection of 86 ticks, with no difference between treatment and control plots (see the electronic supplementary material).

A total of 126 individual tick nymphs and larval pools removed from mammals were tested for infection with *Rickettsia* species, of which 34 (27.0%) tested positive (electronic supplementary material, table S4). Most rickettsial sequences had high homology to species regarded as endosymbionts (*n* = 27; electronic supplementary material, table S4). A total of seven *A. maculatum* samples were infected with the human pathogen *R. parkeri* (1.4% prevalence in larvae and 6.7% prevalence in nymphs). The proportion of ticks infected with *R. parkeri* was not different between treatment and control plots (*p* > 0.05). A total of 83 tick samples were tested for infection with *Borrelia* species of which *B. lonestari* was found in a single *A. maculatum* nymph on an APCNWR treatment plot (electronic supplementary material, table S4).

## 4. Discussion

The invasion of red imported fire ants in the southern United States has had large, negative consequences on ecological communities (reviewed in [13]). We observed decreased small mammal abundances in the presence of *S. invicta* (figure 1), possibly associated with direct (e.g. predation) and indirect effects (e.g. changes in habitat selection and avoidance behaviour) [7,8]. Furthermore, we observed that increased small mammal populations on *S. invicta*-suppressed plots were associated with an increased abundance of on-host ticks (figure 2), consistent with host population regulation of tick populations [14]. Our data suggest that *S. invicta* reduce small mammal populations that, in turn, regulate local tick populations. Thus, these invasive ants may influence tick abundance by affecting the behavioural or physiological mechanisms that control the number of ticks on host individuals, although tick populations may also be influenced directly by *S. invicta* predation. However, the collection of off-host ticks by drag sampling, which was largely restricted to the adult life stage, was not significantly different between control and treatment plots (see the electronic supplementary material). Notably, our study did not investigate other potentially important hosts that support ticks at the larval and nymph stage (i.e. small ground passerines), or adult-stage ticks (i.e. larger mammals), which may also affect tick abundance. It is possible that lower small mammal abundance could increase the frequency of ticks feeding on alternative hosts, including humans, thus increasing disease risk (e.g. [15]).

The cascading effects of *S. invicta* on native small mammal and tick populations have important potential implications for the transmission of tick-borne pathogens, which represent significant public health concerns. Small mammals such as *S. hispidus*, which was heavily parasitized in this study, are reservoirs for numerous tick-borne pathogens including those in the genera *Borrelia*, *Rickettsia*, *Anaplasma* and *Babesia*, as well as multiple viruses [16]. Increased small mammal and tick abundance in *S. invicta*-suppressed areas are expected to intensify contact rates between ticks and hosts, facilitating pathogen transmission. Indeed, increasing host abundance is one of the main drivers of tick-borne disease emergence [17]. Higher tick loads on *R. fulvescens* on treatment plots directly increase vector-host ratios, potentially resulting in increased tick-borne pathogen transmission [18].

The only known human pathogen we detected in ticks removed from mammals was *R. parkeri*, which was present in 1.4% of larvae and 6.7% of nymphs. *Rickettsia parkeri* is a spotted fever group *Rickettsia* long associated with *A. maculatum* and recently associated with human disease in the United States [19]. Although the apparent prevalence of *R. parkeri* infection in our study is low compared with recent research in Virginia (27–55% prevalence; [20]), these studies examined adult ticks located on the northern edge of the *S. invicta* invasion. It is unknown how the current pathogen community in rodent-associated ticks compared with that which occurred in the area prior to *S. invicta* invasion, and the spatial and temporal scale of the contemporary experimental suppression of *S. invicta* may not be sufficient to detect any alteration in pathogen infection associated with a reduction in ant numbers.

While *S. invicta* have pervasive impacts on the ecosystems they invade [16], including depressing populations of endangered taxa [12], land managers need to consider the incidental effects that *S. invicta* suppression may have on tick-borne disease dynamics in some systems. Our work implies that during its invasion *S. invicta* may have produced ecosystem cascading effects that could lead to decreased vector, host and pathogen abundance.

## Supplementary Material

Electronic Supplemental Material

## Data accessibility

Additional information is in the electronic supplementary material. Data are available from the Dryad Digital Repository: http://dx.doi.org/10.5061/dryad.6968k [21]. Tick and pathogen sequences are available on GenBank (http://www.ncbi.nlm.nih.gov/genbank/; KX772750-KX772751 and KX772752-KX772759, respectively).
