# Supplementary material for: Decreased small mammal and on-host tick abundance in association with invasive red imported fire ants (Solenopsis invicta)
Source: Biol Lett. 2016 Sep;12(9):20160463. doi: 10.1098/rsbl.2016.0463 (PMC5046925; doi:10.1098/rsbl.2016.0463)
Supplement: Electronic Supplemental Material [file rsbl20160463supp1.docx]

**Electronic Supplemental Material**

Decreased small mammal and on-host tick abundance in association with invasive red imported fire ants (*Solenopsis invicta*)

Adrian A. Castellanos*, Matthew C. I. Medeiros*, Gabriel L. Hamer, Michael E. Morrow, Micky D. Eubanks, Pete D. Teel, Sarah A. Hamer, Jessica E. Light

*these authors contributed equally

Journal: *Biology Letters*

**Additional methods**

*Analysis of RIFA treatment efficacy*

Treatment plots at Attwater Prairie Chicken National Wildlife Refuge (APCNWR) and the private ranch in Goliad County, Texas, United States (GRR) were chemically treated with Extinguish Plus^TM^ (Central Life Sciences, Schaumburg, IL) for RIFA suppression as part of an existing management plan for Attwater’s prairie chicken [1]; control plots were not treated. At APCNWR, this chemical treatment has been applied annually to the treatment plots since 2009 [1]. Treatment plots at GRR were treated in the falls of 2011 and 2012 [1], and March 2013. Treatment and control plots across both sites ranged in size from 118.6 to 307.6 ha. No treatment plot was directly adjacent to a control plot at either site and plots were separated from each other by at least 1 km. Extinguish Plus^TM^ is a commercially available fire ant bait that is attractive to foraging fire ant workers, is very effective at suppressing fire ant densities, and has little to no impact on non-target arthropods including ticks and native ants [1, 2]. Fire ant activity at APCNWR was assessed by surveys described and published in Morrow et al. [1]. In this study, hot dog slices were left in sites within treatment and control plots for up to 60 minutes. The number of RIFA on hot dog slices was estimated by increments of 10 individuals, up to a maximum of 100 individuals per station. Given the artificial threshold of the upper limit of 100 individuals, we used these published data and analyzed estimated RIFA counts with an ordinal regression (cumulative link mixed model) implemented in the ordinal package in program R. The ordinal levels of estimated counts consisted of 0, ≥1 but ≤ 25, ≥26 but ≤50, ≥51 but ≤75, ≥76 but ≤99, and 100 (or greater). Sampling date (in 2013 and 2014) was added as a random intercept.

*Mammal species richness and diversity*

Diversity and richness of small mammal and tick species in treatment and control plots at each site were assessed with the Shannon-Weaver diversity index (H), including effective species richness (e^H^), using the vegan package in R version 3.22 [3,4]. In these diversity assessments, a high value of H represents a diverse and equally distributed community whereas a value of 0 represents a community with a single species; e^H^ represents the number of species available in equal abundance necessary to reach a particular value of H. We used a null model approach to assess whether the difference in H between plot types was larger than expected by chance. For each site, we calculated the proportions of all species in the total sample. We used these proportions as a probability distribution to simulate 100,000 samples separately for treatment and control plots at both sites. Each simulated sample retained the same sample size as the actual sample. We estimated the Shannon-Weaver diversity index for each simulated community, and took the difference in the indices between the observed and simulated control and treatment plots. We then compared the actual, observed difference in H to the distribution of differences based on the simulated communities. Significance was assumed to be the proportion of simulated differences that were greater than or equal to the observed difference in diversity indices between control and treatment plots.

*Mammal population estimations*

All small mammals captured during this study were handled humanely in accordance with the Texas A&M Animal Care and Use Committee (Permit #2012-100). Mammal species population estimates for treatment and control plots were calculated using our monthly capture and recapture data and the Chapman modification to the Lincoln-Peterson estimation method [5]. Due to the presence of some months with more than two trapping events (July and August 2013 at APCNWR), the Schnabel estimation method was used to better estimate multiple trapping events for these months [6]. Ninety-five percent confidence intervals were generated by using the standard error and FSA package [7] in R. We created estimates for each species by month across the entire sampling period to help curtail the possible effects of recruitment, emigration, and predation on population size. Since many species were captured sparingly over the study, only the species that were frequently captured (*Sigmodon hispidus*, *Baiomys taylori*, and *Reithrodontomys fulvescens* at APCNWR and *B. taylori* and *R. fulvescens* at GRR; table 1) were used in these estimates to avoid large gaps in estimates over time.

*Tick Identification and Pathogen Assessment*

Ticks found on captured mammals were removed, identified based on morphologic and molecular features [8,9], and stored in 70% ethanol. On-host ticks were tested for infection with microbes in the genera *Rickettsia* and *Borrelia* by amplification of the *gltA* [10] and *ompA* genes [11] for *Rickettsia* and 16S–23S rRNA intergenic spacer region [12] for *Borrelia.* Sanger DNA sequencing was performed (Eton Biosciences, San Diego, CA) and sequences were compared to a national database (NCBI Blast) to afford species-level identification. Association between pathogen infection of ticks and *S. invicta* treatment was tested with a Fisher exact test. Larval pools, larval individuals, and nymphs were analyzed in separate contingency tables.

*Drag sampling for off-host ticks*

Off-host tick presence was assessed by sampling questing ticks with a 1-m^2^ piece of corduroy cloth, which was dragged across the vegetation within each treatment and control plot [13]. Drag sampling occurred monthly during the same mammal trapping site visits, weather conditions permitting, across two line transects located in between the trapping transects for a total of 400-m per month. Drag cloths were examined every 20-m for ticks which were removed and placed in 70% ethanol. We used general linear mixed models (GLMM) assuming a negative binomial error distribution to analyze host-seeking ticks found during drag sampling. We used a zero-inflated (ZI) full model if it fit the data better (i.e., lower AIC) than the same model that did not account for zero-inflation. All models were implemented in program R (version 3.2.2) in the package glmmADMB (version 0.8.3.2; [14]). The site (2 levels, APCNWR and GRR) and the season (4 levels, spring = March to May; summer = June to August; fall = September to November; winter = December to February) were added to models as random effects, and treatment was included as the sole fixed effect in the model. Sampling effort was estimated as the distance of drag sampling and input into models analyzing the counts of ticks sampled on drag cloths using the offset function to control for different drag lengths. Significance of all treatment coefficients was assessed through a log-likelihood ratio test of nested models assuming a Chi-square distribution.

**Additional results and discussion**

*Analysis of RIFA treatment efficacy*

Our analysis demonstrated that the odds of being in a lower count category relative to higher categories increased 28.9-fold for treatment relative to control plots (β= -3.4; 95% confidence intervals for regression β = -2.9, -3.9). Only 10% of counts conducted in control areas had no RIFA present. In contrast, 80% of counts conducted in treatment areas had no RIFA present (table S1). These analyses confirm a strong reduction in RIFA populations on treatment plots. Since these results and the results from Morrow et al. [1] strongly conclude that the treatment was efficacious, we did not perform additional tests during our fieldwork on our small mammal trapping transects. Data from the assessment of RIFA activity at GRR were not available. However, a reduction in RIFA activity was noted in Morrow et al. [1]. Therefore we assume that results were similar between APCNWR and GRR.

*Mammal species richness and diversity*

Small mammal species richness was similar at APCNWR and GRR, with seven species at each site (table 1). At APCNWR, control plots had higher richness and diversity with seven species (H = 1.356; e^H^ = 3.880), compared to four species on treatment plots (H = 0.991; e^H^ = 2.694). The proportion of differences between control and treatment plots of simulated APCNWR samples that were equal to or greater than the observed difference was less than 10^-5^, suggesting the difference was not expected by sampling error (two-tailed comparison). Species richness was equivalent at GRR with six species at both treatment and control plots, but diversity indices showed higher diversity at treatment plots (H = 1.203; e^H^ = 3.331) relative to control plots (H = 0.938; e^H^ = 2.557). The proportion of differences between control and treatment plots of simulated GRR samples that were equal to or less than the observed difference was 0.04, greater than the threshold for a two-tailed comparison with an α-level of 0.05. No consistent differences in species diversity were observed between treatment and control plots across sites.

Given the known, negative direct and indirect effects of RIFA on small mammals, we predicted that mammal richness and diversity would be higher on treatment plots compared to control plots. However, we found these measures were inconsistent across sites. Richness and diversity were significantly higher for control plots at APCNWR, and richness was equal yet diversity was higher in treatment plots at GRR. These somewhat conflicting results are likely due to habitat differences among the plots. For example, both of the control plots (but not treatment plots) at APCNWR were located within close proximity to water sources, where only *Oryzomys palustris*, *Cryptotis parva*, and *Peromyscus leucopus* were captured (table 1), in accordance with their habitat preferences [15]. Notably, RIFA have been present at our two study sites for decades (since ca. 1969 and 1979 at APCNWR and GRR, respectively; [16]). This long period of co-occurrence may have resulted in small mammal species adapting to RIFA such that they could still occupy their preferred habitats while avoiding ant encounters. Supporting this point, we captured all small mammal species that were expected to occur in these areas given our trapping methods and habitat [15].

*Mammal population estimations*

Trends in population sizes for each species at APCNWR were similar between treatment and control plots for most of the months during the study (figure S1). Notably, all three species showed larger population sizes at the beginning of the study at APCNWR (June 2013 until October 2013) relative to the following months with peaks of 109, 364.5, and 29 for *S. hispidus* (September 2013), *B. taylori* (August 2013), and *R. fulvescens* (June 2013), respectively (figure S1). Trends in population sizes for *B. taylori* and *R. fulvescens* at GRR also were similar between treatment and control plots over the course of the study, with populations of both species peaking at 131 (February 2014) and 17.7 (April 2014) individuals, respectively (figure S1). It should be noted that population estimates for *B. taylori* may be overestimated due to this species being rarely recaptured during the study (only 13 total recaptures over both sites), which is likely the cause for high capture numbers in August 2013 at APCNWR.

A seasonal influence appeared to cause decreased mammal captures at both plot types in the spring (table 1, figure S1). At APCNWR, all species show increased population sizes in the summer and early fall, with most dropping off after October. This seasonal variability in all captures is likely the result of nearly half (44%) of the mammals captured being *S. hispidus* (mostly from APCNWR; table 1), which featured a dramatic drop in captures during the spring (figure S1). *Sigmodon hispidus* is known to breed nearly year round (February-November; [17]), but is known to have cyclical population increases when resources are ideal for growth [18,19]. In fact, a steep population increase was reported elsewhere in southeast Texas that coincided with the period of higher captures for this species [20]. Decreased captures of *S. hispidus* in the spring were likely the result of the population crashing due to lack of sufficient resources. At GRR, *B. taylori* and *R. fulvescens* showed increased population sizes in the late winter and early spring, likely coinciding with reproductive peaks for these species in the spring [21,22]. Although Smith et al. [13] reported that RIFA disproportionately affected *B. taylori* during times of reproductive activity, this effect, if present, was overwhelmed by the presence of *S. hispidus* and is only hinted at in the population estimates (figure S1).

*Tick Identification and Pathogen Assessment*

All tick morphological and molecular identifications were in agreement. A total of 126 individual tick nymphs and larval pools removed from mammals were tested for infection with *Rickettsia* species, of which 34 (27.0%) tested positive (table S2). Most rickettsial sequences had high homology to species regarded as endosymbionts of unknown pathogenicity to humans and animals (n=27; table S2). A total of seven *A. maculatum* samples were infected with the human pathogen *R. parkeri*, including 2 of 52 larvae pools containing 140 larvae (1.4% prevalence) and 5 of 74 nymphs (6.7% prevalence). *Rickettsia parkeri*-infected ticks were removed from *C. hispidus* and *S. hispidus* from both APCNWR treatment and control plots, including two infected nymphs from the same *C. hispidus* individual. The proportion of larval pools, larval individuals, and nymphs infected with *R. parkeri* was not different between treatment and control plots (*P* > 0.05). The human disease caused by *R. parkieri* includes fever, arthralgias, myalgias, and rash and can be fatal; its diagnosis is often confused with Rocky Mountain spotted fever disease, caused by *R. rickettsii* [24]. A total of 83 tick samples were tested for infection with *Borrelia* species of which *B. lonestari* was found in a single *A. maculatum* nymph removed from a *S. hispidus* on an APCNWR treatment plot (table S2). This *Borrelia* species was once hypothesized to be the etiologic agent of southern tick-associated rash illness, but research does not support this etiology [25].

*Drag sampling for off-host ticks*

Drag sampling totaled 9,400 m^2^ at APCNWR treatment plots, 9,600 m^2^ at APCNWR control plots, and 5,600 m^2^ each at GRR treatment and control plots. A total of 86 off-host ticks were collected, including 74 from APCNWR and 12 from GRR (table S3). Nearly all ticks were adult *A. maculatum* (98.8% of the total off-host ticks) with the exception of one nymphal *I. scapularis* (1.2%) on a GRR treatment plot. At APCNWR, the drag sampling data were highly skewed: approximately half of all off-host ticks were collected from one control plot in August 2013. Ticks collected from the rest of the study period were equally distributed across treatment and control plots. The number of ticks collected by dragging did not vary between treatment and control plots (*P* ≈ 1.0, negative binomial model, random = site, season).

Although these off-host ticks offer little data in terms of understanding what effect RIFA may have on them, if any, they can help draw conclusions about the seasonal phenology of these populations. Texas *A. maculatum* populations are considered coastal populations, which have the highest adult activity in the late summer, although there is some variability in this seasonal phenology [26]. In comparison, inland populations have their highest adult activity in late spring through early summer. The populations of *A. maculatum* found at these sites follow the coastal phenology similar to other Texas populations (table S3), and also some plasticity with several adult ticks being found in non-summer months [26]. Additionally, the nymphs and larvae found on mammals support this coastal phenology with the highest times of activity occurring from late summer until early winter (table S4).

**Supplementary references**

1. Morrow, M. E., Chester, R. E., Lehnen, S. E., Drees, B. M. & Toepfer, J. E. 2015 Indirect effects of red imported fire ants on Attwater’s prairie-chicken brood survival. *J. Wildl. Manage.* **79**, 898–906. (doi:10.1002/jwmg.915)

2. Kaplan, I. & Eubanks, M. D. 2005 Aphids alter the community-wide impact of fire ants. *Ecology* **86**, 1640–1649. (doi:10.1890/04-0016)

3. R Core Team 2015 R: a language and environment for statistical computing.

4. Oksanen, J., Blanchet, F. G., Kindt, R., Legendre, P., Minchin, P. R., O’Hara, R. B., Simpson, P. S., Stevens, M. H. H. & Wagner, H. 2015 vegan: community ecology package.

5. Mares, M. A., Streilein, K. & Willig, M. 1981 Experimental assessment of several population estimation techniques on an introduced population of eastern chipmunks. *J. Mammal.* **62**, 315–328. (doi:10.2307/1380708)

6. Greenwood, J. J. D. & Robinson, R. A. 2006 General census methods. In *Ecological Census Techniques* (ed W. J. Sutherland), pp. 87–185. New York: Cambridge University Press.

7. Ogle, D. H. 2016 FSA: Fisheries Stock Analysis.

8. Sonenshine, D. E. 1979 Ticks of Virginia (Acari, Metastigmata). Virginia Polytechnic Institute and State University, Blacksburg, VA.

9. Beati, L. & Keirans, J. E. 2001 Analysis of the systematic relationships among ticks of the genera *Rhipicephalus* and *Boophilus* (Acari: Ixodidae) based on mitochondrial 12S ribosomal DNA gene sequences and morphological characters. *J. Parasitol.* **87**, 32–48. (doi:10.1645/0022-3395(2001)087[0032:AOTSRA]2.0.CO;2)

10. Williamson, P. C., Billingsley, P. M., Teltow, G. J., Seals, J. P., Turnbough, M. A. & Atkinson, S. F. 2010 *Borrelia*, *Ehrlichia*, and *Rickettsia* spp. in ticks removed from Persons, Texas, USA. *Emerg. Infect. Dis.* **16**, 441–446. (doi:10.3201/eid1603.091333)

11. Zhang, L., Jin, J., Fu, X., Raoult, D. & Fournier, P. E. 2006 Genetic differentiation of Chinese isolates of *Rickettsia sibirica* by partial *ompA* gene sequencing and multispacer typing. *J. Clin. Microbiol.* **44**, 2465–2467. (doi:10.1128/JCM.02272-05)

12. Bunikis, J., Garpmo, U., Tsao, J., Berglund, J., Fish, D. & Barbour, A. G. 2004 Sequence typing reveals extensive strain diversity of the Lyme borreliosis agents *Borrelia burgdorferi* in North America and *Borrelia afzelii* in Europe. *Microbiology* **150**, 1741–1755. (doi:10.1099/mic.0.26944-0)

13. Falco, R. C. & Fish, D. 1992 A comparison of methods for sampling the deer tick, *Ixodes dammini*, in a Lyme disease endemic area. *Exp. Appl. Acarol.* **14**, 165–173. (doi:10.1007/BF01219108)

14. Skaug, H., Fournier, D., Bolker, B., Magnusson, A. & Nielsen, A. 2015 Generalized linear mixed models using ‘AD Model Builder’.

15. Schmidly, D. J. 2004 *The Mammals of Texas*. 6th edn. Austin, TX: University of Texas Press.

16. Callcott, A.-M. A. & Collins, H. L. 1996 Invasion and range expansion of imported fire ants (Hymenoptera: Formicidae) in North America from 1918-1995. *Florida Entomol.* **79**, 240–251. (doi:10.2307/3495821)

17. Cameron, G. N. 1977 Experimental species removal: Demographic responses by *Sigmodon hispidus* and Reithrodontomys fulvescens. *J. Mammal.* **58**, 488–506. (doi:10.2307/1379997)

18. Haines, H. 1963 Geographical extent and duration of the cotton rat, *Sigmodon hispidus*, 1958-1960 fluctuation in Texas. *Ecology* **44**, 771–772. (doi:10.2307/1933026)

19. Grant, W. E., Carothers, P. E. & Gidley, L. A. 1985 Small mammal community structures in the postoak savanna of east-central Texas. *J. Mammal.* **66**, 589–594. (doi:10.2307/1380943)

20. Rodriguez, J. E., Hamer, S. A., Castellanos, A. A. & Light, J. E. 2015 Survey of a rodent and tick community in East-Central Texas. *Southeast. Nat.* **14**, 415–424. (doi:10.1656/058.014.0301)

21. Raun, G. G. & Wilks, B. J. 1964 Natural history of *Baiomys taylori* in southern Texas and competition with *Sigmodon hispidus* in a mixed population. *Texas J. Sci.* **16**, 28–49.

22. Joule, J. & Cameron, G. N. 1980 Demographic studies of *Sigmodon hispidus* and *Reithrodontomys fulvescens* (Rodentia). *Am. Midl. Nat.* **103**, 47–58. (doi:10.2307/2425037)

23. Smith, T. S., Smith, S. A. & Schmidly, D. J. 1990 Impact of fire ant (*Solenopsis invicta*) density on northern pygmy mice (*Baiomys taylori*). *Southwest. Nat.* **35**, 158–162. (doi:10.2307/3671537)

24. Paddock, C. D. 2005 *Rickettsia parkeri* as a paradigm for multiple causes of tick-borne spotted fever in the western hemisphere. *Ann. N. Y. Acad. Sci.* **1063**, 315–326. (doi:10.1196/annals.1355.051)

25. Wormser, G. P. et al. 2005 Microbiologic evaluation of patients from Missouri with erythema migrans. *Clin. Infect. Dis.* **40**, 423–8. (doi:10.1086/427289)

26. Teel, P. D., Ketchum, H. R., Mock, D. E., Wright, R. E. & Strey, O. F. 2010 The Gulf Coast tick: A review of the life history, ecology, distribution, and emergence as an arthropod of medical and veterinary importance. *J. Med. Entomol.* **47**, 707–722. (doi:10.1603/ME10029)

**Figure S1**. Monthly population estimates for treatment and control plot populations of A) *S. hispidus*, B) *B. taylori*, and D) *R. fulvescens* at Attwater Prairie Chicken National Wildlife Refuge (APCNWR) and C) *B. taylori* and E) *R. fulvescens* at a private ranch in Goliad Country, Texas (GRR). Ninety-five percent confidence intervals are shown for each bar. Number of individuals estimated in the population is shown on the y-axis, with the x-axis detailing the study months. APCNWR was sampled from June 2013 until September 2014, while at GRR trapping occurred from October 2013 until July 2014, with the exception of January 2014. The population estimate for *B. taylori* in August 2013 at APCNWR (asterisk) is actually 364.5 individuals with a 95% confidence range of 111 to 711 individuals.

**Figure S1**

**Table S1**. RIFA activity by ordinal count categories of the number of RIFA individuals on hot dog bait slices on control and treatment plots at Attwater Prairie Chicken National Wildlife Refuge.

|  | **Absent** | **≥1 but ≤ 25** | **≥26 but ≤50** | **≥51 but ≤75** | **≥76 but ≤99** | **≥100** |
| --- | --- | --- | --- | --- | --- | --- |
| **Control** | 14 | 6 | 14 | 9 | 26 | 70 |
| **Treatment** | 215 | 8 | 10 | 10 | 15 | 11 |

**Table S2**. Identification and prevalence of pathogens detected in ticks from Attwater Prairie Chicken National Wildlife Refuge (APCNWR) treatment and control plots. *Rickettsia* pathogens were examined in 140 individual larvae (which comprised 52 larval pools) and 74 nymphs. *Borrelia* pathogens were examined in 119 individual larvae (which comprised 42 larval pools) and 40 nymphs.

| **Tick Species and Life State** | **Plot Type** | **No. tested for *Rickettsia*** | **No. infected (%)** | | | | **No. tested for *Borrelia*** | **No. infected (%)** |
| --- | --- | --- | --- | --- | --- | --- | --- | --- |
|  |  |  | Endosymbiont of *A. maculatum*; Candidatus *R. andeanae* | *R. bellii* | *Ixodes scapularis* endosymbiont | *R. parkeri* |  | *Borrelia lonestari* |
| *Amblyomma maculatum* |  |  |  |  |  |  |  |  |
| Larval pool | Treatment | 40 | 7 (17.5) |  |  | 1 (2.5)* | 32 |  |
|  | Control | 12 | 4 (33.3) |  |  | 1 (8.3)* | 10 |  |
| Nymph | Treatment | 45 | 10 (22.2) |  |  | 2 (4.4) | 29 | 1 (3.4) |
|  | Control | 28 | 4 (14.3) | 1 (3.6) |  | 3 (10.7) | 10 |  |
| *Ixodes scapularis* |  |  |  |  |  |  |  |  |
| Nymph | Treatment | 1 |  |  | 1 (100) |  | 1 |  |
| **Total** |  | **126** | **25 (19.8)** | **1 (0.8)** | **1 (0.8)** | **7 (5.6)** | **82** | **1 (1.2)** |

*These particular larval pools consisted of one individual each.

**Table S3**. Monthly on-host captures of larval and nymphal ticks from small mammals, with ticks collected from drag sampling in parentheses. Dashes represent months that were not trapped and NA represents months that were not drag sampled. All ticks were identified as *Amblyomma maculatum* expect two nymphal *I. scapularis*, one collected from *Baiomys taylori* (August 2013) and one collected on a GRR treatment plot.

| **Month** | **APCNWR Treatment** | **APCNWR Control** | **GRR Treatment** | **GRR Control** | **Total** |
| --- | --- | --- | --- | --- | --- |
| June 2013 | 0 (0) | - | - | - | **0 (0)** |
| July 2013 | 1 (0) | 0 (1) | - | - | **1 (1)** |
| August 2013 | 75 (12) | 1 (39) | - | - | **76 (51)** |
| September 2013 | 4 (NA) | 5 (NA) | - | - | **9 (NA)** |
| October 2013 | 14 (NA) | 22 (NA) | 5 (3) | 0 (3) | **41 (6)** |
| November 2013 | 14 (NA) | 5 (NA) | 0 (1) | 0 (1) | **19 (2)** |
| December 2013 | 5 (0) | 2 (0) | 15 (4) | 3 (0) | **25 (4)** |
| January 2014 | 11 (0) | 1 (0) | - | - | **12 (0)** |
| February 2014 | 0 (2) | 0 (0) | 7 (0) | 1 (0) | **8 (2)** |
| March 2014 | 4 (NA) | 13 (NA) | 5 (NA) | 0 (NA) | **22 (NA)** |
| April 2014 | 0 (1) | 1 (0) | 9 (NA) | 0 (NA) | **10 (1)** |
| May 2014 | 1 (0) | 12 (0) | 0 (0) | 0 (0) | **13 (0)** |
| June 2014 | 3 (0) | 0 (0) | 0 (0) | 0 (0) | **3 (0)** |
| July 2014 | 15 (0) | 0 (0) | 0 (0) | 0 (0) | **15 (0)** |
| August 2014 | 3 (6) | 0 (5) | - | - | **3 (11)** |
| September 2014 | 4 (1) | 6 (7) | - | - | **10 (8)** |
| **Total** | **154 (22)** | **68 (51)** | **41 (8)** | **4 (4)** | **267 (86)** |

**Table S4**. Monthly on-host tick captures by life stage (LL = larva, NN = nymph) for each of the four mammal species parasitized by ticks in this study. Dashes indicate that a mammal species was not caught during that month, and the asterisk indicates the one nymphal *Ixodes scapularis* collected from *Baiomys taylori*. All other ticks were identified as *Amblyomma maculatum*.

| **Month** | ***S. hispidus*** | | ***R. fulvescens*** | | ***B. taylori*** | | ***C. hispidus*** | | **Total** | |
| --- | --- | --- | --- | --- | --- | --- | --- | --- | --- | --- |
|  | **LL** | **NN** | **LL** | **NN** | **LL** | **NN** | **LL** | **NN** | **LL** | **NN** |
| June 2013 | 0 | 0 | 0 | 0 | 0 | 0 | 0 | 0 | **0** | **0** |
| July 2013 | 1 | 0 | 0 | 0 | 0 | 0 | - | - | **1** | **0** |
| August 2013 | 44 | 29 | 0 | 0 | 1 | 1* | 0 | 0 | **45** | **30** |
| September 2013 | 0 | 9 | 0 | 0 | 0 | 0 | 0 | 0 | **0** | **9** |
| October 2013 | 36 | 0 | 5 | 0 | 0 | 0 | 0 | 0 | **41** | **0** |
| November 2013 | 5 | 1 | 13 | 0 | 0 | 0 | 0 | 0 | **18** | **1** |
| December 2013 | 7 | 1 | 11 | 0 | 0 | 0 | 6 | 0 | **24** | **1** |
| January 2014 | 2 | 7 | 0 | 0 | 0 | 0 | - | - | **2** | **7** |
| February 2014 | 0 | 0 | 6 | 0 | 0 | 0 | 0 | 0 | **6** | **0** |
| March 2014 | 0 | 7 | 1 | 3 | 0 | 0 | 0 | 6 | **1** | **16** |
| April 2014 | 0 | 2 | 1 | 0 | 1 | 1 | - | - | **2** | **3** |
| May 2014 | 0 | 12 | 0 | 0 | 0 | 1 | - | - | **0** | **13** |
| June 2014 | 0 | 3 | 0 | 0 | 0 | 0 | 0 | 0 | **0** | **3** |
| July 2014 | 0 | 3 | 0 | 0 | 0 | 0 | - | - | **0** | **3** |
| August 2014 | 0 | 2 | 0 | 0 | 0 | 0 | - | - | **0** | **2** |
| September 2014 | 2 | 7 | 0 | 0 | 0 | 0 | - | - | **2** | **7** |
